# Supplementary material for: MetaRibo-Seq measures translation in microbiomes
Source: Nat Commun. 2020 Jun 29;11:3268. doi: 10.1038/s41467-020-17081-z (PMC7324362; doi:10.1038/s41467-020-17081-z)
Supplement: Supplementary file 10 — Supplementary Data 7 [file 41467_2020_17081_MOESM10_ESM.zip › File2/Confidence_High_Taxonomy/178676_out.krona.html]

Javascript must be enabled to view this page.

members
magnitude
magnitudeUnassigned
count
unassigned
taxon
rank

178676\_out

189

superkingdom
189
2

phylum
4
976

200643
4
class

3
171549
4
order

SRS044535\_contig\_number\_13989SRS048870\_contig\_number\_564SRS075341\_contig\_number\_18971

2005525
1
family

375288
1
genus

species
387661
1

SRS893253\_contig\_number\_109

phylum
1239
185

class
909932
4

order
909929
1

family
1843491
1

1
970
genus

713030

SRS143885\_contig\_number\_contig-100\_95.218174
1
species

1843489
3
order

family
3
31977

genus
2
906

species
187326
2

SRS046496\_contig\_number\_16371SRS077916\_contig\_number\_11441

genus
29465
1


SRS105074\_contig\_number\_13848
1
29466
species

91061
119
class

order

SRS021477\_contig\_number\_contig-100\_215.178542
119
1
186826

1300
88
family

genus
88
1301

5
1
1303
species

SRS019794\_contig\_number\_contig-100\_122.123

subspecies

SRS074682\_contig\_number\_30297SRS078434\_contig\_number\_121
2
1891914

1458253
2

SRS054196\_contig\_number\_contig-100\_10.120670SRS143360\_contig\_number\_10971
subspecies

species
1311

SRS045763\_contig\_number\_10289SRS075947\_contig\_number\_29304SRS104539\_contig\_number\_49233SRS146878\_contig\_number\_49726
4

species group
671232
1

species
1328
1

SRS021496\_contig\_number\_30435

species

SRS016225\_contig\_number\_20710
1
1739371

species

SRS011343\_contig\_number\_17170SRS012983\_contig\_number\_19147SRS013170\_contig\_number\_contig-100\_141.298178SRS013949\_contig\_number\_36652SRS015158\_contig\_number\_contig-100\_12.147390SRS015692\_contig\_number\_23107SRS015755\_contig\_number\_contig-100\_42.78333SRS015895\_contig\_number\_contig-100\_126.18985SRS015985\_contig\_number\_10727SRS015989\_contig\_number\_9648SRS017304\_contig\_number\_3166SRS018145\_contig\_number\_21857SRS018359\_contig\_number\_contig-100\_70.39278SRS018573\_contig\_number\_contig-100\_81.154040SRS018612\_contig\_number\_contig-100\_30.22915SRS019128\_contig\_number\_41396SRS019798\_contig\_number\_contig-100\_195.96342SRS022536\_contig\_number\_51751SRS024144\_contig\_number\_20227SRS024447\_contig\_number\_contig-100\_179.201666SRS044230\_contig\_number\_contig-100\_27.203242SRS045197\_contig\_number\_51207SRS053584\_contig\_number\_contig-100\_77.82575SRS053917\_contig\_number\_46597SRS054569\_contig\_number\_contig-100\_96.62853SRS055378\_contig\_number\_51464SRS063215\_contig\_number\_19136SRS064449\_contig\_number\_41886SRS064937\_contig\_number\_20066SRS075353\_contig\_number\_contig-100\_39.171410SRS076874\_contig\_number\_18430SRS077441\_contig\_number\_contig-100\_386.58179SRS097918\_contig\_number\_contig-100\_7.137693SRS098735\_contig\_number\_36383SRS101280\_contig\_number\_14100SRS104165\_contig\_number\_36009SRS104519\_contig\_number\_49885SRS104521\_contig\_number\_25826SRS104539\_contig\_number\_30634SRS104711\_contig\_number\_28093SRS104968\_contig\_number\_contig-100\_20.109877SRS105121\_contig\_number\_5418SRS143678\_contig\_number\_contig-100\_166.110611SRS143684\_contig\_number\_29010SRS143728\_contig\_number\_contig-100\_124.17620SRS144014\_contig\_number\_17243SRS144096\_contig\_number\_9041SRS144124\_contig\_number\_contig-100\_148.352387SRS144144\_contig\_number\_12006SRS144621\_contig\_number\_contig-100\_70.411051SRS146769\_contig\_number\_2914SRS146878\_contig\_number\_34016SRS146996\_contig\_number\_24025SRS147106\_contig\_number\_58743SRS147281\_contig\_number\_contig-100\_497.118047SRS147386\_contig\_number\_contig-100\_84.256836SRS147442\_contig\_number\_12411SRS148773\_contig\_number\_651SRS148777\_contig\_number\_17936SRS149488\_contig\_number\_contig-100\_190.191SRS893229\_contig\_number\_12831SRS893318\_contig\_number\_contig-100\_760.146380SRS893328\_contig\_number\_contig-100\_300.97403
63
1313

species

SRS143729\_contig\_number\_contig-100\_10.10SRS143756\_contig\_number\_2SRS150034\_contig\_number\_4447
3
257758

45634
1

SRS146830\_contig\_number\_8547
species

1305

SRS016746\_contig\_number\_contig-100\_53.196605SRS018394\_contig\_number\_22330SRS020406\_contig\_number\_22782SRS023841\_contig\_number\_12238SRS042307\_contig\_number\_1390SRS043729\_contig\_number\_16128SRS063672\_contig\_number\_105
7
species

species
28037

SRS147338\_contig\_number\_6489
1


SRS049147\_contig\_number\_30727
1
1579343
species

1419814

SRS058730\_contig\_number\_contig-100\_55.180090
1
species

3
33958
family

genus
3
1578

species
3

SRS065099\_contig\_number\_contig-100\_478.163338SRS074976\_contig\_number\_contig-100\_315.277044SRS1055086\_contig\_number\_12265
1598

family
25
81852

1350
25
genus

1352

SRS013951\_contig\_number\_8613SRS046712\_contig\_number\_701SRS143466\_contig\_number\_contig-100\_700.178651
3
species

species
1351
22

SRS013098\_contig\_number\_48600SRS013638\_contig\_number\_19444SRS015264\_contig\_number\_23081SRS016018\_contig\_number\_contig-100\_609.103623SRS018313\_contig\_number\_12023SRS018575\_contig\_number\_8106SRS020328\_contig\_number\_contig-100\_1672.219298SRS020869\_contig\_number\_39242SRS024009\_contig\_number\_10243SRS053356\_contig\_number\_26125SRS056519\_contig\_number\_15767SRS063985\_contig\_number\_contig-100\_352.174956SRS064757\_contig\_number\_20345SRS074586\_contig\_number\_contig-100\_1233.42006SRS075821\_contig\_number\_contig-100\_125.139365SRS077231\_contig\_number\_22347SRS098717\_contig\_number\_19768SRS104693\_contig\_number\_12244SRS140492\_contig\_number\_25281SRS142503\_contig\_number\_55465SRS142599\_contig\_number\_32118SRS147557\_contig\_number\_31147

186827
2
family

2
46123
genus

species
2

SRS063485\_contig\_number\_61710SRS078738\_contig\_number\_36610
1581061

class
62
186801

62
186802
order

family
1
186804

genus
1870884
1

species
1496
1

SRS045645\_contig\_number\_34827

1898207

SRS098073\_contig\_number\_5543
1
species

species

SRS047014\_contig\_number\_37289SRS146813\_contig\_number\_29456
2
1897045

29
186803
family

genus
1164882
24

936595
23

SRS015038\_contig\_number\_10697SRS016133\_contig\_number\_24024SRS016969\_contig\_number\_contig-100\_162.226400SRS018923\_contig\_number\_17917SRS018923\_contig\_number\_17917SRS023595\_contig\_number\_55650SRS042589\_contig\_number\_7253SRS043461\_contig\_number\_16017SRS043461\_contig\_number\_16017SRS050628\_contig\_number\_8260SRS064423\_contig\_number\_25308SRS076868\_contig\_number\_32223SRS078003\_contig\_number\_contig-100\_597.170653SRS097895\_contig\_number\_contig-100\_59.214965SRS098818\_contig\_number\_16309SRS100027\_contig\_number\_14294SRS142896\_contig\_number\_30211SRS144237\_contig\_number\_18542SRS144300\_contig\_number\_contig-100\_878.251663SRS148150\_contig\_number\_contig-100\_343.152989SRS148284\_contig\_number\_12713SRS149280\_contig\_number\_17333SRS893291\_contig\_number\_5315
species

species
1

SRS065542\_contig\_number\_contig-100\_157.110491
467210

species
1

SRS048981\_contig\_number\_contig-100\_54.57072
658087

1506553
1
genus

208479

SRS893289\_contig\_number\_contig-100\_307.47621
1
species

genus
1
189330

species
88431

SRS019496\_contig\_number\_10420
1

1
28050
genus

species
446043

SRS049402\_contig\_number\_17656
1

genus
1
33042

1

SRS063190\_contig\_number\_15286
410072
species

family
541000
21

genus
204475
1

species

SRS075878\_contig\_number\_23944
1
745368

genus
2
292632

species
2302959
1

SRS143598\_contig\_number\_21110

species
2302962

SRS014412\_contig\_number\_21227
1

1

SRS024075\_contig\_number\_16534
552398
species

1
1935176
genus

species
1871018
1

SRS046369\_contig\_number\_16899

genus
2
216851

853
2

SRS021219\_contig\_number\_11957SRS049712\_contig\_number\_25052
species

genus

SRS020400\_contig\_number\_5716SRS893230\_contig\_number\_11231
14
1263
2


SRS023715\_contig\_number\_27666SRS043001\_contig\_number\_contig-100\_1491.70168SRS052027\_contig\_number\_contig-100\_643.122437SRS056273\_contig\_number\_contig-100\_855.204215SRS075987\_contig\_number\_27800SRS104314\_contig\_number\_contig-100\_326.107016SRS1055035\_contig\_number\_contig-100\_1195.76894SRS143616\_contig\_number\_contig-100\_1046.293115SRS148514\_contig\_number\_contig-100\_161.138365SRS148820\_contig\_number\_contig-100\_295.150640
10
2293228
species

1

SRS013687\_contig\_number\_27879
1930000
species

1

SRS149325\_contig\_number\_14285
165186
species

family
3
216572

genus
459786
3

species
876091

SRS014459\_contig\_number\_contig-100\_214.236256SRS075021\_contig\_number\_contig-100\_1601.54849SRS148196\_contig\_number\_40412
3

3
31979
family

580596
1
genus

species
1

SRS149075\_contig\_number\_12352
2292003

1485
2
genus

1

SRS042643\_contig\_number\_8654
2293028
species

species
2292206
1

SRS049446\_contig\_number\_8009

1

SRS015794\_contig\_number\_11188
1897048
species

1897047

SRS104975\_contig\_number\_24082
1
species
